# Words Matter: How Attorney Language Abstraction and Emotional Valence Shape Juror Decision-Making

**DOI:** 10.3390/bs15101355

**Published:** 2025-10-04

**Authors:** Justice Healy, Monica K. Miller, Yueran Yang

**Affiliations:** 1 Interdisciplinary Social Psychology Ph.D. Program, University of Nevada, Reno, NV 89557, USA; 2 Department of Sociology, University of Nevada, Reno, NV 89557, USA; mkmiller@unr.edu; 3 Department of Psychology, University of Nevada, Reno, NV 89557, USA; yuerany@unr.edu

**Keywords:** language abstraction, emotional valence, jury decision-making, persuasion, courtroom communication, liability

## Abstract

The language used by attorneys at trial could influence case outcomes, impacting fairness and wrongful convictions. At trial, attorneys choose their words to manage impressions the jury forms of the defendant, thereby influencing case outcomes. This study examines whether the abstraction and emotional valence of attorneys’ language at trial influence jurors’ decision-making. In this 2 × 2 factorial experiment, 273 online participants read an attorney’s closing statement regarding a civil case, with the emotional valence of the attorney’s descriptions being either positive or negative, and the abstraction concrete or abstract (e.g., a negative–concrete description being “the cost of removing these *cancer-causing* chemicals is *millions of dollars*” vs. the corresponding abstract description, “the cost of removing these *health-hazardous* chemicals is *enormous*”). The results revealed that attorney language abstraction and emotional valence influenced jurors’ perceptions of the case: Participants judged the defendant as more liable when exposed to negative–concrete language than positive–concrete language—a difference not present with abstract language. Findings suggest that attorneys might benefit from tailoring their language in closing arguments and that jurors’ decisions can be influenced by *how* information is conveyed—highlighting implications for courtroom communication and legal outcomes.

## 1. Introduction

*“For it is not enough to know what we ought to say; we must also say it as we ought.”*—

Aristotle (n.d.)

The role of attorneys is to defend their client’s best interests. Previous legal research captures the importance of closing arguments, emphasizing that they are a lawyer’s “final and often most powerful opportunity to shape jurors’ views of the case” (Wallace, 1995). At trial, attorneys know that they must be convincing to the jury, who is charged with reaching a verdict based on evidence—yet it seems possible that the way in which evidence is linguistically presented matters as much as the evidence itself.

Little research has focused on the impact of lawyers’ language use on jurors, despite the fact that case outcomes are not only shaped by the strength of the evidence but also by the persuasiveness of the lawyers who present the case (O’Barr, 1985; Visher, 1987). This paper examines the effect of language abstraction in the courtroom, a topic that has received little empirical attention. Specifically, we investigate how lawyers’ use of abstract versus concrete language affects jurors’ legal judgments when evaluating positive or negative descriptions of a case.

### 1.1. Language Abstraction

Language abstraction refers to the degree to which something is described in abstract terms (‘Leah is *aggressive*’) or concrete terms (‘Leah *punched* Claire’) as defined by the linguistic category model (Semin & Fiedler, 1988). Concrete words refer to things, events, and other characteristics that can be perceived through human senses (e.g., *trees*, *walking*, *red*), and abstract words refer to ideas that are not immediately perceived through the senses (e.g., *economics*, *calculating*, *disputable*; Neuman et al., 2012).

On a broader level, social cognition researchers provide theoretical explanations for how language plays a critical role in matching social cognition with social reality. Semin (2000) explains that social communication is meant to turn cognition into action, arising from both internal factors (e.g., information processing) and external factors (e.g., social stimuli). In other words, cognition becomes intention for an action, and language is used as the means of implementing the action. Therefore, language is an “implementational device” for cognition (Semin, 2000).

Along these lines, language abstraction can be used to pursue social goals. Some examples include starting and ending romantic relationships; evaluating individual and group decisions; pursuing political and gender power; and persuading others (Rubini et al., 2017). Furthermore, a describer’s use of language abstraction influences how message recipients evaluate a message and make judgments about the describer’s attitude and goals regarding the person described (i.e., the target; Douglas & Sutton, 2006). When a describer uses *positive*, abstract descriptions, recipients infer that the describer is friends with and biased in favor of the target; when a describer uses *negative*, abstract descriptions, recipients infer that the describer and target have an unfavorable relationship (Douglas & Sutton, 2006).

Beyond social cognition research, language abstraction influences decision-making. Namely, abstract/concrete paradoxes arise in decision-making contexts when a problem elicits differing responses based on whether it is framed abstractly or concretely, as described by Struchiner et al. (2020). For example, experimental philosophy studies have found that individuals tend to say that moral responsibility is incompatible with determinism when a problem is presented *abstractly*, yet judge that a deterministic agent who commits a *concrete*, heinous crime (e.g., killing his family) can be fully morally responsible (Cova et al., 2012). Similarly, in behavioral economics, people are more responsive to the suffering of a single identifiable victim than to abstract groups or statistical victims (Kogut & Ritov, 2005).

Parallel effects arise in legal and moral cognition: people endorse deterrence-based theories of punishment framed *abstractly*, but they assign punishment based on retributive instincts in *concrete* cases (Kneer & Machery, 2019; Donelson & Hannikainen, 2020). Furthermore, legal theorists have emphasized how abstract language in court contributes to juror decision-making. Feigenson (2000) argues that abstraction can subtly guide jurors’ interpretations of guilt, responsibility, and moral blame—noting that this “linguistic shaping of legal blame” is critical for how jurors evaluate and recall narratives. Taken together, these findings underscore that language abstraction profoundly influences how people evaluate moral, legal, and social responsibility across contexts.

### 1.2. Language Abstraction and Emotional Valence

**Figure 1:**
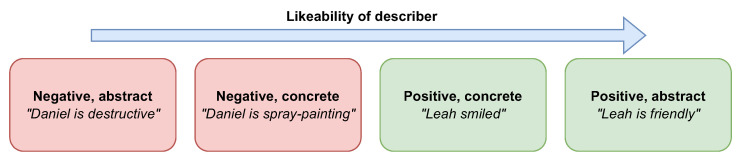
Describer’s Likability Based on Emotional Valence and Abstraction of Language, According to Douglas and Sutton (2010).

Past research has shown that language abstraction and emotional valence can interactively influence perceptions. For example, Douglas and Sutton (2010) found that, when describers talk about others’ negative actions, the describers are perceived as less likable when they use abstract language (‘Daniel is *destructive*’), and more likable when they use concrete language (‘Daniel is *spray-painting*’). In contrast, impressions of describers were more positive when they described others’ positive behaviors abstractly as opposed to concretely (see Figure 1). Douglas and Sutton (2010) found a significant interaction between emotional valence and language abstraction for audiences’ perceptions of the likability of a describer.

Audiences have greater liking for speakers who describe others positively rather than negatively (Wyer et al., 1990), and a positive or negative liking of something influences decision-making (Damasio, 1994). These communication phenomena have implications in court, where attorneys make statements that are inherently positive or negative. The dichotomous quality of descriptive language is unlikely to evade the attention of an audience (Maass et al., 1989) such as a jury.

For attorneys, there are many potential avenues to influence the way jurors perceive them and case outcomes through language abstraction and emotional valence. For example, attorneys can describe negative behaviors concretely and positive behaviors abstractly. For both prosecution and defense, there are instances in which it is appropriate to use positive descriptions. For example, the defense attorney in the O.J. Simpson case stated the following positive, abstract words (italicized) to the jury in closing: “You are *empowered* to do *justice*”; “One of my *favorite* people in history is the *great* Frederick Douglass”; and “I’d like to comment and to *compliment* [the prosecutors] on what I thought were *fine* arguments” (Linder, 1995). These statements indicate the many ways attorneys can manipulate their language abstraction and emotional valence.

One way that attorneys may aim to make a favorable impression, then, is by increasing the frequency with which they use positive descriptions—particularly when referring to their own client. This strategy must be applied judiciously, as excessive positivity may appear disingenuous, and it might furthermore be complicated in court, where both sides’ attorneys must often make negative statements to frame evidence. However, while attorneys cannot always avoid negative statements, they can manipulate their use of concrete or abstract descriptions.

Scholars of legal rhetoric agree that attorneys’ statements in court are sites of substantial narrative construction. Attorneys use language to frame events and characters in ways that significantly shape juror interpretations (Amsterdam & Hertz, 1992), and beyond merely recounting facts, such legal storytelling actively organizes meaning and moral judgment for jurors (Amsterdam & Bruner, 2009). Lawyers can thus shape their language towards various ends, and we set out to examine the more subtle linguistic factors at play in lawyers’ framing of a case. While previous research indicates language abstraction and emotional valence interactively influence the impressions that listeners form of describers, what remains unanswered is how this applies to impressions that jurors form of attorneys and legal cases.

### 1.3. Attorney Language and Juror Perceptions

Attorney language influences jurors’ perceptions of their credibility. Research suggests that when attorneys rely on abstract, emotionally neutral communication—often to appear objective or professional—they may undermine their perceived trustworthiness (Durant & Leung, 2016; Lambert, 2022). This is because attorneys’ use of emotionally restrained language can lead jurors to perceive them as overly detached or unfeeling, reducing their persuasive impact (Lambert, 2022). In court, a message’s emotional weight impacts jurors’ interpretations of information and the assignment of responsibility (K. Edwards & Bryan, 1997). Therefore, although neutrality might seem like attorneys’ safest approach, it might fail to engage jurors on a human level.

Additionally, in courtroom communications, attorneys often use legalese, which is technical but often convoluted legal language that is abstract, complex, and emotionally neutral (Martínez et al., 2023). Excessively technical language tends to reduce juror perceptions of attorney confidence and clarity (Chapman, 1993). In fact, both laypeople and legally trained individuals tend to respond more positively to attorneys who use clear, jargon-free language, perceiving them as more credible and relatable (Martínez et al., 2023). In addition, attorneys’ emotional expression, when it aligns with the context of the case, can enhance juror perceptions of their sincerity and investment in the case outcome (Lambert, 2022). In fact, attorneys who use emotionally resonant language are often perceived as more credible than those who default to abstract, emotionally flat styles of communication (Lambert, 2022; Chapman, 1993). Furthermore, attorneys’ use of assertive, concrete language is associated with jurors viewing them as more persuasive (Chapman, 1993). Thus, a *concrete* and *emotionally attuned* approach from attorneys might influence juror decisions in their favor.

### 1.4. Prosecution Versus Defense Language

On the other hand, some studies link *abstract* attorney language to juror judgments favorable to the attorney’s case, and research indicates the prosecution and defense use language abstraction differently. Two studies by Schmid and Fiedler (1998) examined whether subtle, linguistic attribution cues in the courtroom impacted mock jurors’ verdicts and punishments. Attorneys-in-training gave closing speeches as the defense and prosecution. Using abstract language, prosecutors made attributions to defendants, and defense attorneys made attributions to the victim. They found that attorneys using abstract, negative, and dispositional language (e.g., “He is *aggressive*”) increased juror guilt attributions and harsher sentences; using concrete, neutral, or situational language (e.g., “He *shouted*”) led to jurors reducing blame and punishment.

Additionally, a content analysis of the Nuremberg trials indicated that defense attorneys described their clients abstractly and positively, whereas prosecutors used more interpretive action verbs and negative descriptions to indicate defendant responsibility (Schmid & Fiedler, 1998). Finally, Burguet (2011) manipulated linguistic abstraction using passages from a newspaper article about a rape crime, finding that more linguistic abstraction (vs. concreteness) was associated with more severe guilt judgments by mock jurors.

Despite the large body of research investigating how attorneys can be more persuasive, a topic that remains uninvestigated is the effect of lawyers’ language abstraction on juror perceptions of their credibility or case outcomes. Understanding how language abstraction affects juror impressions has implications for attorney communication training, legal advocacy strategies, and reducing bias in courtroom decision-making.

### 1.5. Research Overview

This experiment investigated how language abstraction and emotional valence in attorneys’ communications of a legal case influence jurors’ decision-making. Specifically, we conducted a mock trial experiment in which we manipulated a plaintiff attorney’s language abstraction and the emotional valence of their descriptions.

The experiment presented a civil case scenario that described a company being sued for leaking chemicals into a woman’s drinking water, allegedly causing her to develop breast cancer. Mock jurors read an overview of the case and then a closing statement in which the plaintiff’s attorney described the defendant using either positive or negative *abstract* or *concrete* descriptions (see Appendix A). Afterward, participants answered questions about their liability judgments and verdicts. They also answered questions about the attorney’s credibility and their perceptions of the plaintiff and defendant.

### 1.6. Hypotheses and Research Questions

We hypothesized that attorney language abstraction (abstract vs. concrete) and emotional valence (positive vs. negative) would interact to influence jurors’ judgments about liability and verdict, attorney credibility, and the plaintiff and defendant.

**Research Question: Liability Perceptions**

Will attorney language abstraction and emotional valence significantly influence liability ratings and verdict?

**Hypothesis** **1.** *Attorney Credibility and Language.*

Based on previous findings regarding the impact of language abstraction and valence on likability (Douglas & Sutton, 2010), we predict that language abstraction and valence will interact, such that jurors’ ratings of attorney credibility will increase in the following order: negative–abstract, negative–concrete, positive–concrete, and positive–abstract.

**Hypothesis** **2.** *Perceptions of Plaintiff and Defendant.*

Attorney language valence will impact juror perceptions of plaintiff and defendant:

**Hypothesis** **2a.** *Jurors who read negative (vs. positive) descriptions of the defendant will rate the plaintiff as more honest and deserving of sympathy*.

**Hypothesis** **2b.** *Jurors who read negative (vs. positive) descriptions of the defendant will rate the defendant as less ethical, trustworthy, and knowledgeable about wrongdoing*.

## 2. Method

### 2.1. Participants

We recruited 310 participants. To control for data quality, the following participants were excluded from analysis (*n* = 39): those who completed the survey in an unusually short amount of time (defined as three standard deviations below the sample’s average completion time), failed to pass one or more attention checks, or demonstrated nonsensical or inattentive open-ended survey responses.

The final sample consisted of 273 participants (48.0% female, mean age = 31.8 years) from Amazon Mechanical Turk (MTurk; *n* = 152, 58.6%, *M*age = 38.15 years) and a mid-sized university in the western U.S. (*n* = 121, 41.4%, *M*age = 23.0 years). University students were recruited either via email or the university’s SONA system; MTurk participants were recruited through CloudResearch. Participants were recruited from both MTurk and the university to increase this study’s power, and they were evenly distributed across experimental conditions. All participants were U.S. citizens over 18 years of age.

A priori power analysis using G*Power 3 (Faul et al., 2007) indicated a minimum sample of 180 to detect a medium effect (*d* = 0.25) with 80% power (*α* = 0.05). We recruited beyond this to account for potential exclusions due to inattention or bot responses.

### 2.2. Design

This study used a 2 (language abstraction: concrete, abstract) × 2 (language valence: positive, negative) between-subjects factorial design to test the impact of attorney language abstraction and valence on jurors’ case perceptions, perceptions of attorney credibility, and verdicts.

### 2.3. Materials and Measures

#### 2.3.1. Civil Case Summary

Participants read a civil case summary in which a woman sues a chemical company for $500,000 in compensatory damages for leaking chemicals into her drinking water and thus allegedly causing her cancer. The civil case summary included expert witness testimony from two expert witnesses, one from the plaintiff and defense. The civil case summary and expert testimony can be found in Appendix A. The expert witness testimony was adapted from C. P. Edwards (2022) and modeled on depositions and testimony by forensic toxicologists and scientists.

#### 2.3.2. Closing Statements

Participants read one of the four versions of the plaintiff attorney’s closing statement in which the attorney described the chemical company. The four versions of the closing statements reflected the combination of the attorney’s linguistic valence and abstraction manipulations: concrete and positive, concrete and negative, abstract and positive, and abstract and negative. In other words, the plaintiff attorney *concretely* or *abstractly* described the chemical company in either *positive* or *negative* terms.

For example, in the negative emotional valence condition, one concrete description was “the cost of removing these *cancer-causing* chemicals is *millions of dollars*, and it will probably take *decades*” and the corresponding abstract description was “the cost of removing these *health-hazardous* chemicals is *enormous*, and it will probably take *a very long time*.” The closing statements can be found in Appendix A.

#### 2.3.3. Manipulation Check

After reading the closing statement, participants rated the emotional valence (ranging from negative to positive) and linguistic abstraction (ranging from concrete to abstract) of the closing statement they read on scales of 1 to 7.

#### 2.3.4. Liability Form

Participants rated the likelihood that the defendant caused the plaintiff’s cancer on a scale of 0 (least likely) to 100 (most likely), and they indicated how much money the plaintiff should be awarded after finding, or imagining that they found, the defendant liable. Participants also rendered a verdict (liable vs. not liable).

#### 2.3.5. Perceptions of the Defendant

Participants rated their perceptions of the defendant’s ethicality and trustworthiness. Participants also rated the likelihood that the defendant knowingly contaminated the water supply. They reported these perceptions on scales of 1 (*not at all*) to 7 (*extremely*).

#### 2.3.6. Perceptions of the Plaintiff

Participants rated the plaintiff’s honesty and their sympathy for the plaintiff on scales of 1 (*not at all*) to 7 (*extremely*).

#### 2.3.7. Attorney Credibility Scale

Participants completed the Attorney Credibility Scale (ACS; Ziemke & Brodsky, 2015, adapted from Brodsky et al., 2010) to report their perceptions of the plaintiff attorney’s credibility. The ACS uses 20 items with a 10-point Likert scale, leading to scores for domains of confidence, likability, trustworthiness, and knowledge (Brodsky et al., 2010). This scale was designed to evaluate witness credibility, but such constructs could also be perceived in attorneys by jurors (Ziemke & Brodsky, 2015) and hence suits this study. The ACS asks mock jurors to rate the attorney on a 10-point scale with antonym adjectives on either side (e.g., honest—dishonest). In the current study, the 20-item Attorney Credibility Scale demonstrated excellent internal consistency (Cronbach’s α = 0.94). For conceptual clarity, likability was defined by items such as *Friendly*, *Respectful*, *Kind*, *Mannered*, and *Pleasant*; trustworthiness included *Trustworthy*, *Truthful*, *Dependable*, *Honest*, and *Reliable*; confidence was assessed via *Confident*, *Articulate*, *Relaxed*, *Poised*, and *Self-assured*; and knowledge comprised *Informed*, *Logical*, *Educated*, *Wise*, and *Scientific*.

#### 2.3.8. Attention Checks

To ensure participants’ attentiveness, we included three attention checks throughout the survey. Two items assessed recall of core case information (e.g., “What is the name of the woman suing the company?”). A third item was a simple instruction (“Please choose D to continue”).

#### 2.3.9. Demographic Questionnaire

A demographic questionnaire was used at the end of this study to collect information about participants’ race, gender, and income.

### 2.4. Procedure

Participants could choose to participate in this study based on reading a brief recruitment description of this study posted on MTurk, the university SONA system, and sent via email to students. After clicking on the link to the survey, participants were informed of the risks and benefits of the research and then could consent to participate.

Participants read a civil case summary in which a woman sues a chemical company for leaking chemicals into her drinking water and allegedly causing her cancer. The civil case summary included expert witness testimony from both the plaintiff and defense.

Participants were then randomly assigned to one of four cells produced by a 2 (language abstraction: concrete, abstract) × 2 (language valence: positive, negative) between-subjects factorial design. Participants read the plaintiff attorney’s closing statement in which the attorney described the chemical company. The closing statements reflected each condition of the attorney’s linguistic valence and abstraction: *positive–concrete*, *positive–abstract*, *negative–concrete*, and *negative–abstract.* After reading a case summary and one of the four closing statements (see Appendix A), participants quantitatively rated its emotional valence and abstraction.

The dependent variables were mock jurors’ verdicts (liable vs. not liable) and perceptions of the following (rated numerically): the plaintiff attorney’s credibility; the likelihood that the defendant caused the plaintiff’s cancer; the defendant’s liability, ethicality, trustworthiness, and knowledge of water contamination; and the plaintiff’s honesty, deservingness of sympathy, and how much money they should be awarded.

Finally, participants completed a demographic questionnaire to assess the similarity of the sample’s demographic characteristics to those of the U.S. population, and to determine if any individual characteristics (e.g., political orientation, gender) moderate the relationship between the independent and dependent variables.

### 2.5. Analysis Plan

We conducted a series of factorial ANOVAs, regression analyses, and exploratory follow-ups to test our hypotheses and research question. Specifically, we used a 2 (language abstraction: concrete, abstract) × 2 (language valence: positive, negative) between-subjects design to assess the effects of attorney language on jurors’ liability ratings, verdicts, and perceptions of attorney credibility. Binary verdicts were analyzed using logistic regression, while continuous dependent variables were analyzed using ANOVA or linear regression. Manipulation checks and sample effects were evaluated and reported; sample source (UNR vs. MTurk) was excluded from final analyses due to non-significance. 

## 3. Results

We first screened the data and excluded the following participants (*n* = 37): (a) those whose completion time was three standard deviations below the average, (b) those who failed to pass one or more attention checks, and (c) those who demonstrated nonsensical or inattentive survey responses—responses that did not make sense at the level of spelling, diction, or syntax, or responses that were unrelated to the survey. This resulted in a final analytic sample of 273 participants.

We conducted a series of analyses to examine how attorney language abstraction and emotional valence influenced jurors’ liability ratings, verdicts, perceptions of attorney credibility, and perceptions of the plaintiff and defendant. Although sample (UNR vs. MTurk) was initially included as a factor, it had no significant effects and was excluded from final analyses. Below, we present the results in order of theoretical and practical importance.

### 3.1. Manipulation Check Results

We conducted manipulation checks to verify whether participants perceived the intended differences in emotional valence and abstraction. For emotional valence, participants in the positive conditions rated the attorney’s language as significantly more positive (*M* = 4.15, *SD* = 1.59) than those in the negative conditions (*M* = 2.27, *SD* = 1.35), *t*(256.4) = 10.43, *p* < 0.001; *d* = 1.27, 95% CI [1.03, 1.51], indicating the valence manipulation was successful. In contrast, participants’ ratings of language abstraction did not significantly differ between abstract (*M* = 4.85, *SD* = 1.37) and concrete (*M* = 5.03, *SD* = 1.28) conditions, *t*(270.9) = 1.12, *p* = 0.26; *d* = 0.14, 95% CI [−0.10, 0.37]. These results suggest that participants were sensitive to the emotional tone of the language but not to its level of abstraction. Nonetheless, language abstraction still had an effect on liability ratings, next reported—so it is possible that the manipulation checks were not sensitive enough to detect the manipulation.

### 3.2. Juror Liability Ratings

**Figure 2:**
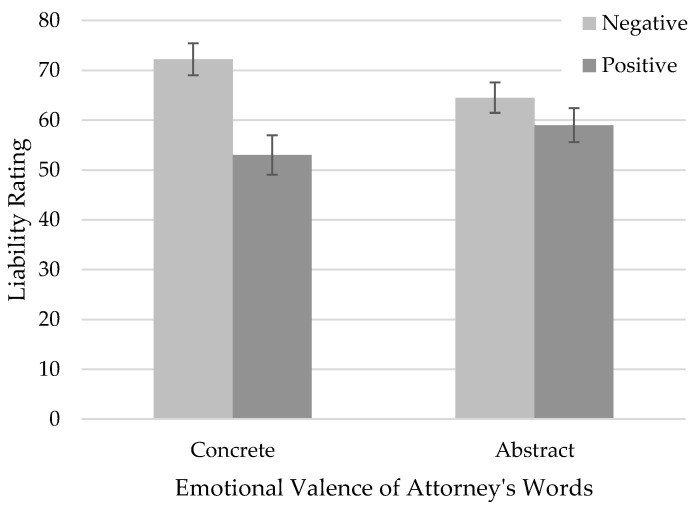
Juror Liability Ratings of Defendant Based on Emotional Valence and Abstraction of Attorney Language. Note. Error bars denote one standard error around the mean.

We first examined the research question of whether attorney language abstraction and emotional valence influenced jurors’ perceptions of the defendant’s liability. We conducted a 2 (abstraction: concrete, abstract) × 2 (valence: positive, negative) factorial ANOVA to examine the effects of language abstraction and emotional valence on jurors’ liability ratings. The results revealed a significant interaction effect between language abstraction and emotional valence, *F*(1, 269) = 4.02, *p* = 0.046, *η*^2^ = 0.014. To probe the interaction, we conducted simple effects analyses using Tukey’s HSD tests (Tukey, 1949). Results showed that when attorneys used *abstract* language, participants’ liability perceptions did not differ between positive statements (*M* = 59.0, *SD* = 27.7) and negative statements (*M* = 64.5, *SD* = 26.2), *t(*269) = 1.15, *p* = 0.25; *d* = 0.20, 95% CI [−0.14, 0.53]. However, when the attorney used *concrete* language, participants’ liability perceptions were significantly higher when they read the negative statement (*M* = 72.2, *SD* = 26.5) than the positive statement (*M* = 53.0, *SD* = 31.9), *t*(269) = 3.93, *p* < 0.001, *d* = 0.65, 95% CI [0.41, 0.89]. The cell means are visualized in Figure 2.

Beyond the significant interaction, the main effect of valence was also significant, *F*(1, 269) = 12.71, *p* < 0.001, *η*^2^ = 0.05. Participants’ liability perceptions were significantly higher when they read the negative statement (*M* = 70.0, *SD* = 26.4) than the positive statement (*M* = 58.9, *SD* = 29.6), *d* = 0.41, 95% CI [0.17, 0.64]. The main effect of language abstraction was not significant, *F*(1, 269) = 0.11, *p* = 0.74, *η*^2^ < 0.001. Overall, these findings provide support for the hypothesis that attorney language abstraction and emotional valence significantly influence liability ratings.

### 3.3. Juror Verdicts

**Figure 3:**
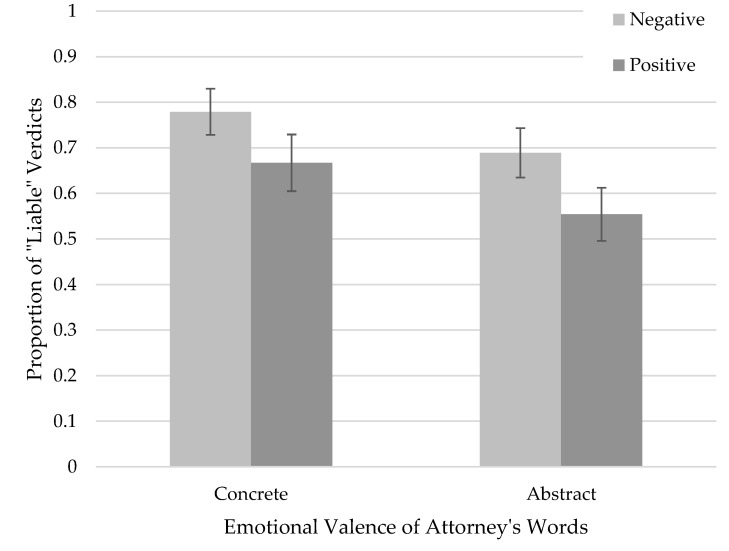
Proportions of Liability Verdicts Based on Emotional Valence and Abstraction of Attorney Language. Note. Error bars denote one standard error around the mean. The number of jurors who rendered a liable verdict varied across conditions.

Next, we examined juror verdicts. The overall percentage of “liable” verdicts was 67.4%. More specifically, we examined our research question on whether the plaintiff’s attorney’s language abstraction and emotional valence influenced binary verdicts (Liable vs. Not liable) using logistic regression. The interaction between valence and abstraction approached significance, $\chi^{2}\left(1\right)=3.22,\;p=0.07$. This result (see Figure 3) trended similarly to juror liability ratings (see Figure 2). This suggests that participants who read positive–concrete statements were somewhat less likely to render a liable verdict compared to those who read negative–concrete statements. Although not conventionally significant, this trend supports the idea that emotional valence might have a greater impact when attorney language is concrete rather than abstract.

Importantly, the main effect of emotional valence was significant, $\chi^{2}\left(1\right)=4.59,\;p=0.03$, indicating that verdicts differed overall between positive and negative statements when collapsing across levels of language abstraction. On the other hand, the main effect of language abstraction was not significant, $\chi^{2}\left(1\right)=0.02,\;p=0.90$, suggesting that abstract versus concrete language did not significantly affect verdicts when collapsing across emotional valence. Taken together, these results suggest that jurors’ liability decisions were influenced more by the emotional tone of the attorney’s language than by its level of abstraction.

### 3.4. Effects of Language on Attorney Credibility

We conducted a 2 × 2 factorial ANOVA to examine how attorney language valence and abstraction influenced jurors’ ratings of attorney credibility (Hypothesis 1). The interaction between valence and abstraction was not statistically significant, *F*(1, 269) = 0.11, *p* = 0.74, *η*^2^ < 0.001. In addition, neither the main effect of valence (*F*(1, 269) = 0.39, *p* = 0.53, *η*^2^ = 0.002) nor the main effect of abstraction (*F*(1, 269) = 0.38, *p* = 0.54, *η*^2^ < 0.001) was significant. Thus, there was no evidence to support the hypotheses that valence and abstraction influenced juror perceptions of attorney credibility.

### 3.5. Effects of Emotional Valence on Perceptions of Trial Actors

Finally, we tested whether the attorney’s emotional valence influenced juror perceptions of the plaintiff and defendant (Hypothesis 2). The analyses revealed no statistical significance. The attorney’s emotional valence did not significantly predict perceptions of the plaintiff’s honesty and deservingness of sympathy (*b* = −0.01, SE = 0.26, *p* = 0.97) or the defendant’s ethics (*b* = −0.13, SE = 0.32, *p* = 0.68). A simple regression analysis revealed no significant effect of emotional valence on juror perceptions of the attorney’s credibility (*b* = −0.13, SE = 0.16, *p* = 0.42). Thus, the hypothesis was not supported.

## 4. Discussion

The current study found that attorney language abstraction and emotional valence interacted to influence jurors’ perceptions of liability. Our results revealed that the effect of emotional valence depended on the level of language abstraction. In the *concrete* condition, participants assigned higher liability when attorneys used negative, compared to positive, language. However, in the *abstract* condition, liability ratings did not significantly differ based on valence. This interaction suggests that emotional framing had a greater impact when attorneys used concrete, specific language, whereas abstract language muted the persuasive effects of emotional tone. Thus, it appears that concrete descriptions were more influential than abstract descriptions on liability perceptions.

Verdicts followed a similar pattern to liability ratings. Participants were most likely to render a liable verdict after reading *negative*–concrete statements, and they were least likely to after reading *positive*–concrete statements. Although not conventionally significant, this pattern supports the idea that concrete, emotionally negative language may have greater persuasive force than abstract language.

### 4.1. Interpretation of Language Abstraction and Valence Effects

We propose a few possible explanations for our findings that *concrete*, but not *abstract*, language drove higher liability ratings when attorneys used negative rather than positive language. One explanation is that concrete descriptions of a defendant might exert greater influence on jurors’ liability judgments than abstract ones. Namely, the language we manipulated addressed the defendant’s character rather than the core trial issue (i.e., whether the defendant caused the plaintiff’s cancer). It is possible that the concrete descriptions were more evocative and noteworthy to mock jurors and thus more impactful on their liability perceptions.

Notably, if jurors relied on character cues when assigning liability, this reflects inappropriate legal reasoning—evaluating the defendant based on personal traits rather than case-relevant evidence. In torts trials, jurors are meant to determine if an injury results from negligence or intentional actions (Cohen, 2009). This would suggest that mock jurors might have made a fundamental attribution error, attributing results of someone’s behaviors to that person’s dispositional qualities and minimizing the situational factors at play (Ross, 1977). Mock jurors perhaps made broader inferences about liability based on impressions of the defendant’s character rather than actions—which is what jurors should be basing their judgments on. In essence, jurors may have been disproportionately swayed by *concrete* character portrayals, leading them to infer blame in a manner inconsistent with legal standards.

Further, we based our study on Douglas and Sutton (2010), which found that language abstraction and emotional valence independently—and interactively—increase the perceived *likability* of a describer in the following order: negative–abstract, negative–concrete, positive–concrete, positive–abstract. We examined this linguistic effect on *liability*. Although abstract, positive language enhanced speaker likability in their research, in our study, *concrete* language drove higher liability ratings when attorneys used negative compared to positive language—but in the *abstract* condition, liability ratings did not significantly differ based on valence.

It is possible that participants perceive making liability judgments as more consequential than making likability judgments. Another possible explanation lies in the contextual expectations of legal proceedings: Jurors may view abstract language as vague, strategic, or even evasive when used by attorneys. In contrast, concrete descriptions may convey credibility, specificity, and a clearer evidentiary foundation, which are highly valued in courtroom settings.

Furthermore, while positive, abstract language may elevate likability in everyday interactions by implying that the describer has positive traits (as Douglas and Sutton noted), in a legal environment it might raise suspicion or feel insufficiently grounded, diminishing its persuasive weight. Thus, although the two studies both investigate language abstraction and valence, the discrepancy in findings might reflect fundamental differences in context and evaluators’ assessments of a describer’s goal.

### 4.2. Attorney Credibility

We initially hypothesized that attorney language abstraction and valence would influence credibility, based on past findings linking abstraction and emotional valence to speaker likability (e.g., Douglas & Sutton, 2010) and linking attorney’s use of concrete language (e.g., Lambert, 2022; Durant & Leung, 2016; Martínez et al., 2023; Chapman, 1993) and emotional expression (e.g., K. Edwards & Bryan, 1997) to more favorable juror impressions. However, no such effects emerged in our data. One possible explanation is that participants viewed the attorney as an advocate for a party in the case, rather than a neutral observer. Jurors might discount positively framed language about a defendant as strategic or disingenuous, thereby dampening any effect on credibility. Thus, the mechanisms by which language affected liability ratings remain to be determined.

### 4.3. Emotional Valence and Defendant Ethics

Interestingly, emotional valence did not significantly influence participants’ perceptions of the defendant’s ethicality, even in the negative–concrete condition that included explicit language about cover-ups and lawsuits. One possible explanation is that both the positive and negative manipulations presented moral signals that were either too vague or not directly diagnostic of the defendant’s ethical behavior, which is what participants were asked to evaluate. Positive descriptions (e.g., collaboration with Greenpeace, affordable pricing) may have seemed irrelevant or insufficiently exonerating, while negative descriptions (e.g., lawsuits, cover-ups) may have felt disconnected from the specific wrongdoing in question.

Another explanation involves the broader framing of the case. All four versions of the attorney’s closing argument concluded with an assertion of liability (“Chemco [the defendant] owes Kathy [the plaintiff] compensation…”), which might have anchored participants’ moral judgments on the idea that harm occurred. In this context, participants might have defaulted to seeing the defendant as unethical across all conditions, reducing variability and producing a potential floor effect. Additionally, our significant interaction effect (of abstraction and valence) on liability suggests that concrete framing played a more decisive role than valence in shaping liability judgments. The concrete language conveyed specific actions (e.g., pollution, lawsuits), which likely had a stronger influence on perceptions of wrongdoing than emotional tone alone. This pattern may help explain why valence had no main effect on ethics ratings. Future research could probe this line of inquiry.

### 4.4. Discussion of Manipulation Check Results

It is of note that our manipulation check results showed that while the emotional valence manipulation was successful, the abstraction manipulation did not significantly affect participants’ perceived abstraction ratings. This aligns with past research suggesting that abstraction is more cognitively subtle and harder for laypersons to detect than concrete ideas (e.g., Semin & Fiedler, 1988). Nonetheless, our theoretical design yielded significant effects of abstraction and valence on liability, suggesting that the manipulation was effective even if the manipulation check did not capture it. Future studies may benefit from alternative manipulation checks—such as linguistic coding or expert validation—to assess abstraction more accurately.

### 4.5. Limitations

#### 4.5.1. Participants and Online Modality

This study has methodological limitations. First, its external validity might be limited by the use of a convenience sample drawn from Amazon Mechanical Turk and a university student pool. These participants might differ in meaningful ways from actual jurors, limiting the generalizability of the findings to real courtroom contexts, though notably, some research indicates that online results are similar to those of traditional, in-person studies (e.g., Gosling et al., 2004). We included manipulation and attention checks to mitigate these concerns. Another potential limitation in this study is that participants were aware they were part of a study, which might have influenced how they processed the information, particularly with respect to the language manipulations.

The online modality of this study also threatens external validity such that, in real trials, jurors hear case arguments presented orally, often accompanied by visual cues such as tone, pace, and body language. In contrast, our participants read written case materials. It is possible that attorney language abstraction and emotional tone would have different effects in an auditory format. Future work could explore how jurors process attorney language in audio or video formats, or within deliberating juries as opposed to deliberating alone, as in our study.

#### 4.5.2. Manipulation

It is also possible that the interaction between abstraction and valence observed in this study was driven more by differences in informational severity than by the linguistic framing itself (an issue noted in other studies of linguistic abstraction in legal cases; see (Schmid & Fiedler, 1998)). For example, our negative–concrete condition included explicit accusations of serious wrongdoing (e.g., destroying documents, concealing pollution test results), while the negative–abstract condition used more generalized language (e.g., “[the defendant was] not upfront”) that lacked those direct allegations. As a result, participants may have reacted more strongly to the morally and legally charged content of the concrete condition rather than to the language abstraction per se. Future research should ensure that manipulations of abstraction and valence are matched for severity and moral content to disentangle the effects of language from the gravity of the behavior described.

This study also faced a construct validity challenge. Manipulating language abstraction—without influencing emotional valence—proved difficult, as more abstract descriptions tend to differ not only in content but also in tone. Defining what constitutes “abstract” or “concrete” language is not without controversy. Studies vary widely in how they differentiate abstraction from concreteness, ranging from general vs. specific, fictional vs. actual, and indetermined vs. determined (Struchiner et al., 2020). Our abstraction and valence manipulations might have been partially confounded.

For example, the negative–concrete condition explicitly accused the defendant of “destroying documents and concealing or lying about the results of their environmental pollution tests,” whereas the negative–abstract version more vaguely stated that the defendant was “not upfront about the results of their environmental pollution tests.” These discrepancies may have unintentionally introduced differing levels of perceived immorality and illegality between conditions. As prior research suggests, jurors may conflate moral blameworthiness with legal responsibility (e.g., Alicke, 1992; Feigenson, 2000), potentially intensifying the persuasive force of the concrete condition not solely due to language abstraction but due to heightened moral outrage. Thus, some effects we attributed to abstraction may instead—or additionally—reflect variation in the perceived severity of the behaviors described.

Future research could further pilot test linguistic manipulations of abstraction and emotional valence. This could help determine to what extent any subsequent effects on participants are due to changes in *informational* content versus changes in the *abstractness* or *emotional valence* of the content, given the inevitability of varying informational content when varying word choice. We acknowledge this to be one of the more pressing obstacles in this type of study and believe future research could clarify it.

Furthermore, our manipulation might have had features that limited its impact on mock jurors’ perceptions of attorney credibility. For one, in our study, the plaintiff’s attorney only described the defendant. Although in real trials, attorneys rarely focus on neutral third parties, experimental designs could use neutral targets to better isolate the effects of language abstraction and valence on perceived credibility. Such a strategy could clarify the causal mechanisms linking language to credibility judgments. Additionally, our study only tested the plaintiff’s closing argument. Including a defense argument would have required additional study materials that may have introduced confounds. Still, future studies could incorporate both plaintiff and defense arguments to yield more ecologically valid trial designs once mechanisms are better understood.

#### 4.5.3. External Validity

Similarly, a realism-related limitation concerns the positive emotional valence conditions. In the positive versions of the closing statement, the *plaintiff’s* attorney highlights the social and environmental benefits of the *defendant’s* product. While this may not reflect typical plaintiff strategies in real tort cases, it was meant to maintain consistency in structure across conditions and ensure a strong manipulation of emotional valence. Although this limits ecological validity, it allowed us to systematically test how valence and abstraction influence jurors. Future research could explore more naturalistic closing arguments that better mirror how attorneys balance praise and criticism in real-world litigation.

A related realism concern is that the negative conditions referenced other lawsuits against the defendant—content that might not be admissible in real trials. While this limits ecological validity, it was included to strengthen the manipulation and reflect persuasive strategies attorneys sometimes attempt. Future work could isolate admissible language to test courtroom-relevant effects more directly.

Another limitation of this study is that the evidentiary strength of the plaintiff’s case was not designed to meet real-world legal thresholds for admissibility. Courts require expert testimony to be based on scientifically reliable methods, and in toxic tort cases, this often includes epidemiological evidence demonstrating a doubling of risk. In our scenario, such evidence was not presented, and the plaintiff’s expert testimony would likely not survive summary judgment. While this raises concerns about ecological validity, our study was designed to examine how jurors respond to different forms of language. By using a simplified but semi-plausible trial scenario, we were able to isolate the effects of language abstraction and emotional valence on juror judgments. Future work should build on this design by incorporating legally robust and admissible evidence.

### 4.6. Intellectual Merit and Broader Impact

This research explores previous findings that audiences tend to prefer speakers who describe others positively rather than negatively (e.g., Wyer et al., 1990), and that this effect is moderated by language abstraction (Douglas & Sutton, 2010), by examining such effects in a mock jury context. While courtroom language has been extensively studied, little to no research has specifically examined the impact of attorneys’ language abstraction on jurors—despite the fact that case outcomes are often shaped by the persuasiveness of the attorneys who present them (O’Barr, 1985). This study addresses this gap. The findings offer insight into how attorneys can more effectively communicate with juries during closing statements, suggesting that concrete descriptions—whether framed positively or negatively—have the strongest influence on juror decision-making.

The implications of this study pertain to how attorney language influences juror decision-making. Our findings indicate that language abstraction and emotional valence can influence how jurors rate a defendant’s liability, and although this study does not speak to the accuracy of juror verdicts or whether specific language styles lead to just outcomes, it highlights the persuasive power of verbal framing on legal decisions.

These findings may inform future research and legal practice by illustrating that seemingly minor variations in attorneys’ closing statements can meaningfully affect jurors’ judgments. While this does not suggest that attorneys should adopt particular rhetorical strategies to manipulate outcomes, it raises important questions about the role of language in shaping perceptions of liability—an issue central to fairness in the courtroom. Future research might extend this work by examining how such effects unfold in real-world legal settings, including live or recorded trial contexts and group deliberations. Finally, these findings can inform future research on persuasion and language across disciplines such as philosophy, psychology, law, politics, business, and education.

## 5. Conclusions

Seemingly overlooked linguistic characteristics of attorneys can impact the outcome of a case. At trial, attorneys choose their words carefully to manage impressions the jury forms of not only the defendant, but also of the attorneys themselves. These impressions can determine whether a defendant is found liable or not liable, guilty or not guilty. This study examined whether the abstraction (concrete vs. abstract) and emotional valence (positive vs. negative) of attorneys’ closing statements influence jurors’ impressions and judgments. In a 2 × 2 factorial experiment, online participants read an attorney’s closing statement regarding a civil case, varying in language abstraction and emotional valence. Results showed that, although jurors’ perceptions of attorney credibility were not significantly affected, language abstraction and emotional valence did shape liability ratings and to a slightly lesser extent, verdicts. These findings suggest that attorneys’ verbal framing of case information can influence outcomes and could inform communication best practices in courtroom advocacy. Future research could examine how these effects operate in real-world courtroom settings, including jury deliberation and spoken delivery, to expand the practical applications of this work.

## Data Availability

Please contact the lead author, Justice Healy (justiceh@unr.edu), for data supporting reported results.

## Appendix A. Case Summary and Closing Statements

**Introduction**

You will now read about a civil lawsuit in which an individual sues a company for allegedly causing her cancer.

In the following pages, you will find:

1. A definition of burden of proof
2. A definition of liability
3. A case overview
4. Testimony from expert witnesses
5. Closing statements
6. Questions about yourself and your perceptions of the case

Please read carefully and do your best on the questions asked.

**Burden of Proof Instructions**

The parties must persuade you, by the evidence presented, that what they are required to prove is more likely to be true than not true. This is referred to as “the burden of proof.”

After weighing all of the evidence, if you cannot decide that something is more likely to be true than not true, you must conclude that the party did not prove it. You should consider all the evidence, no matter which party produced the evidence.

In criminal trials, the prosecution must prove that the defendant is guilty beyond a reasonable doubt. But in civil trials, such as this one, the party who is required to prove something needs to only prove that it is more likely to be true than not true.

**Liability Instructions**

The plaintiff in this case claims that a chemical company, named Chemco, leaked chemicals into her drinking water supply, causing her cancer. Both parties agree to specific facts of the case, such as that the plaintiff has been accurately diagnosed with cancer and Chemco leaked chemicals into her drinking supply. However, they disagree on the plaintiff’s claim that the chemicals leaked into the water caused the plaintiff’s cancer. To establish this claim, the plaintiff must prove that the leaked chemical causes cancer. In other words, the plaintiff must prove that Chemco is liable, or legally responsible, for causing her cancer.

**Case Overview**

**Mrs. Kathy Summers—Plaintiff**
**Chemco Chemicals Inc.—Defendant**
**Dr. Raymond Jones—Plaintiff expert witness**
**Dr. Mark Davis—Defense expert witness**

Kathy, age 32, is suing a large chemical manufacturing company called Chemco. She claims that some of the chemicals that Chemco has stored at a dump one mile from her house have seeped into the neighborhood’s water supply, and that regularly drinking the contaminated water caused her ovarian cancer. It was after moving to her neighborhood that Kathy was diagnosed with ovarian cancer, which she alleges was caused by the Ketamine in the water supply. The company does not dispute the fact that high levels of Ketamine leaked from the chemical dump site into the water supply, but Chemco officials argue that there is no proof that Ketamine is a cancer-causing agent.

**Kathy’s Cancer**

Kathy’s ovarian cancer was detected at a late stage. As a result, she had to have both of her ovaries surgically removed. This operation involved major surgery that required a week-long stay in the hospital. Because both ovaries were removed, Kathy is unable to have children. Her cancer has spread since the surgery, which was two years ago. She has not been able to return to her job as a marketing consultant, and Kathy is frequently in pain.

**Trial Issue**

A major issue at trial is whether or not a particular chemical, called Ketamine, causes cancer. If so, then the chemical company is liable for damages, since both parties accept that high levels of Ketamine leaked from the dump into the neighborhood’s water supply; if not, then the chemical company is not liable.

Kathy is asking for compensatory damages in the amount of $500,000 for medical costs, lost income, pain and suffering.

**Evidence Presented at Trial—Plaintiff**
**Expert Witness for Kathy: Dr. Raymond Jones**

Dr. Jones testifies that Ketamine could potentially cause health problems. He gave female laboratory rats large doses of Ketamine manufactured by one of five different companies, for a period of one year. He then evaluated their health on a variety of measures and compared across different brands. Rats exposed to the defendant’s chemical developed 10 times more health complications than the average. For example, some of the rats developed tumors. Ovarian cancer was not a very common type of health problem overall, but it occurred most often in rats that took the defendant’s Ketamine. Additionally, Dr. Jones also conducted field tests on the water and wildlife found in a lake located next to the dump site. He found that some species of animals had developed deformities and infertility. Dr. Jones tested the water and found the defendant’s Ketamine to be present. He concludes that the defendant’s chemical could lead to similar health problems in humans.

**Evidence Presented at Trial—Defense**
**Expert Witness for Chemco: Dr. Mark Davis**

Dr. Davis testifies that Ketamine in general does not increase the risk of cancer. A national survey of women showed that 10 out of every 1000 women age 30–40 develop ovarian cancer, regardless of what kind of chemicals they are exposed to. Dr. Davis also conducted a study in which he compared women who had been unknowingly exposed to high amounts of one of four different chemical products, one of which was Ketamine, or no chemicals at all. Cancer rates did not differ for women in the different groups. Although he did not specifically compare different brands of Ketamine, he concludes that the defendant’s chemical, one of many brands that women in this study had been exposed to, is unlikely to increase the risk of ovarian cancer.

**Table A1:** Closing Arguments (Randomly Assigned Condition).

**Negative–Concrete Condition:** **Over the past 20 years**, Chemco has released **contaminants** into the water supply and has been sued **six times** in the past 5 years by other women diagnosed with ovarian cancer. The chemical company has also been **sued in seven lawsuits for releasing chemical waste into** the environment.	**Negative–Abstract Condition:** Chemco has **long been known** to **negatively affect** the water supply and has been sued **several times** in the past 5 years by other women suffering from ovarian cancer. The chemical company has also been **accused of harming** the environment.
Greenpeace, an environmental watchdog organization, **reported that the chemical company caused a scientifically significant amount of** pollution in the environment and **tried to cover up these allegations by destroying documents and concealing or lying** about the results of their environmental pollution tests. The cost of removing these **cancer-causing chemicals** from the water supply by Kathy’s house is **millions of dollars**, and it will probably take **decades** to get rid of most of these pollutants.	Greenpeace, an environmental watchdog organization, **has concerns about the company’s extensive contribution** to pollution and **believes that Chemco is not upfront** about the results of their environmental pollution tests. The cost of removing these **health-hazardous chemicals** from the water supply by Kathy’s house is **enormous**, and it will probably take a **very long time** to get rid of most of these pollutants.
**Positive–Concrete Condition:** We acknowledge that Chemco makes over **200** products that **work and are widely used** in people’s **skin care, clothing, and home products**. Their chemical product goes into **hundreds** of products, including cosmetics, carpets, clothes, leather, and textiles. Therefore, their product might be in the clothes you wear, the carpet you walk on, and cosmetics you apply to your skin to **make it soft every day**.	**Positive–Abstract Condition:** We acknowledge that Chemco makes **effective and beneficial** products. Their chemical product goes into **countless** products, including cosmetics, carpets, clothes, leather, and textiles. Therefore, their product might be in the clothes you wear, the carpet you walk on, and cosmetics you **enjoy** on your skin every day.
Millions of people around the world have received benefits from their products such as **affordability and accessibility**. Consumers pay a fair price for everyday items that this widely used product is in. Greenpeace, an environmental watchdog organization, also reported that Chemco was **developing new best practices methods and handbooks** with Greenpeace to **decrease the amount of waste secreted into the environment by 50% in the next 5 years**.	Millions of people around the world have received benefits from their products. Consumers also pay a fair price for everyday items that this widely used product is in. Greenpeace, an environmental watchdog organization, also reported that Chemco was **working together with Greenpeace** to achieve more **environmentally friendly practices in upcoming years.**

Note. Participants did not see any text as bolded.

That being said, Chemco owes Kathy compensation because the Ketamine they leaked into her water supply caused her cancer.
